# Hb Narges Lab, a Novel Hemoglobin Variant of the β-Globin Gene

**DOI:** 10.34172/aim.2022.55

**Published:** 2022-05-01

**Authors:** Mohammad Hamid, Zahra Shahbazi, Bijan Keikhaei, Hamid Galehdari, Alihossein Saberi, Alireza Sedaghat, Gholamreza Shariati, Marziye Mohammadi-Anaei

**Affiliations:** ^1^Department of Molecular Medicine, Biotechnology Research Center, Pasteur Institute of Iran, Tehran, Iran; ^2^Thalassemia & Hemoglobinopathy Research Center, Health Research Institute, Ahvaz Jundishapur University of Medical Sciences, Ahvaz, Iran; ^3^Department of Genetics, Faculty of Sciences, Shahid Chamran University of Ahvaz, Ahvaz, Iran; ^4^Department of Medical Genetic, Faculty of Medicine, Ahvaz Jundishapur University of Medical Sciences, Ahvaz, Iran; ^5^Department of Endocrinology, Ahvaz Jundishapur University of Medical Sciences, Ahvaz, Iran; ^6^Narges Medical Genetics & PND Laboratory, No. 18, East Mihan Ave, Kianpars, Ahvaz, Iran

**Keywords:** β-Thalassemia, Mutation, Iran

## Abstract

In this study, we describe a new missense variant on the β-globin gene in a heterozygous form in a female individual. Standard methods were used to determine red blood cell indices and perform hemoglobin analyses. Molecular studies were performed on the genomic DNA isolated from peripheral blood cells. Beta-globin genes were amplified and sequenced. We report a novel mutation on the β-globin gene (HBB), c.134 C>T; p.S44F variant, in the heterozygote state which was detected in a female of Persian ethnic origin in the Khuzestan province, southern Iran, that we named Hb Narges Lab (HbNL) variant. This mutation was predicted to be disease-causing in all except one *in silico* prediction tools. This variant was reported for the first time worldwide, had no shown hematological abnormalities but should be considered when inherited in the compound heterozygous form with β- thalassemia (β0-thal) carrier, which might result in the phenotype of thalassemia intermedia.

## Introduction

 Thalassemia is considered the most common recessive genetic disease worldwide. β-thalassemia is caused by beta-globin production deficiency or absence. In the healthy carrier, this condition is clinically mild, and routine blood testing can be used to detect it easily. In the patients born with a 25% chance of being afflicted from parents who are healthy carriers, however, this condition is intermediate or severe.^1,2^ Iran is one of the countries where β-thalassemia is prevalent among the Eastern Mediterranean countries. Regarding high consanguinity among the population, two and three million β-thalassemia carriers and 25 000 patients are estimated to exist in Iran.^3,4^ We describe heterozygosity for a new missense variant on the β-globin gene in a female individual of a Persian ethnic origin in the Khuzestan province, southern Iran, who referred for thalassemia carrier detection test.

## Case Report

 During the national screening program in the Khuzestan population, a female, 28 years old of Persian ancestry was referred for the workup of anemia. The study was evaluated and approved by the Ethics Committee of Pasteur Institute of Iran. Standard methods were used to determine red blood cell indices and perform hemoglobin analysis. After obtaining written informed consent, molecular studies were done on the genomic DNA isolated from peripheral blood cells based on a salting-out procedure.^5^ To identify β-thalassemia genotypes, we amplified and DNA sequenced the entire β-globin genes on an ABI PRISMTM 3130 apparatus (Applied Biosystems, Foster City, CA, USA).

**Figure 1:**
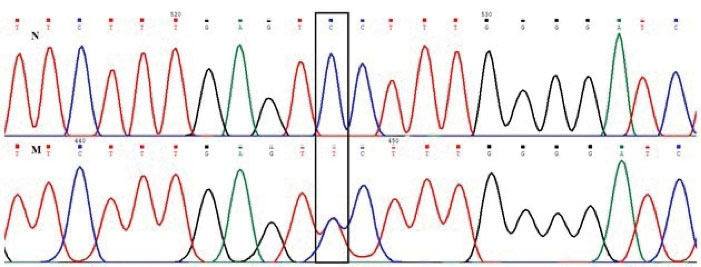
β-Globin Gene Sanger Sequencing (N: Normal allele, M: Heterozygote mutant genotype for HBB: p.S44F variant).

 A novel mutation was found in the coding region of the HBB gene in a female. Table 1 presents the red blood cell indices and the results of Hb analyses. Sequencing of the β-globin gene of the index individual found the c.134 C > T; p.S44F variant in a heterozygote state (Figure 1). This mutation was named Hb Narges Lab (HbNL) variant. To study pathogenicity based on the hematological indices, we regarded the following indices in normal individuals including mean corpuscular volume (MCV) > 80.0 fL, mean corpuscular Hb (MCH) > 27.0 pg, and Hb A2 < 3.5%. HbNL in the heterozygote form had no shown clinical and hematological abnormalities, although the red blood cell indices of this person were slightly lower than normal, indicating mild microcytic anemia.

**Table 1:** Demographic Features and Blood Indices of the Patient

**Parameter**	**Amount**
Gender-Age	F-28
RBC (10^6^/uL)	4.69
MCV (fL)	77.8
MCH (pg)	24.5
MCHC (g/dL)	31.5
Hb (g/dL)	11.5
Hb A (%)	97.3
Hb F (%)	0.3
Hb A2(%)	2.4
α-Genotype	αα/αα
β- Genotype	c.134 C > T; p.S44F

**Figure 2:**
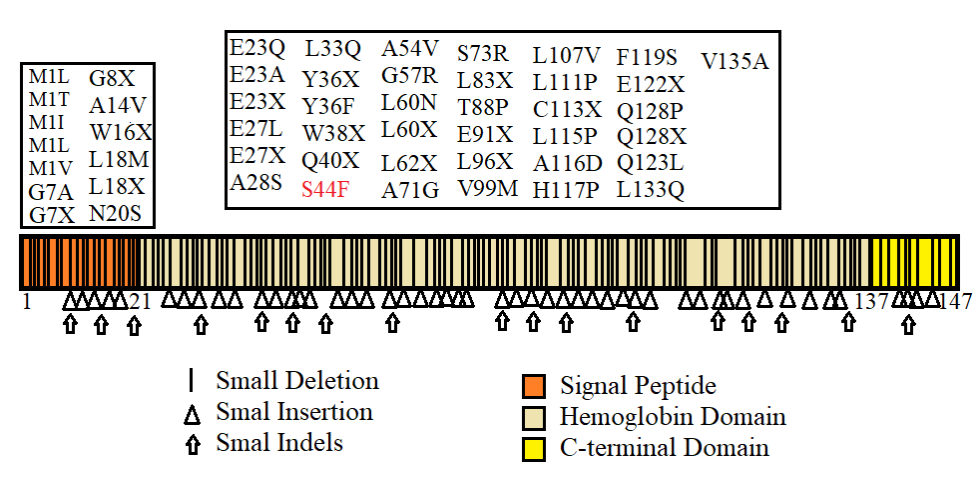
Schematic Representation of the β-Globin Protein and its Reported Mutations. Our novel variation is marked in red.

**Figure 3:**
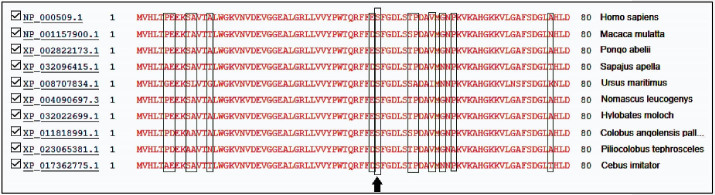
Conserved Interspecies Amino Acids in β-Globin Proteins. Our novel variation changes a conserved amino acid which is shown by the black arrow.

**Figure 4:**
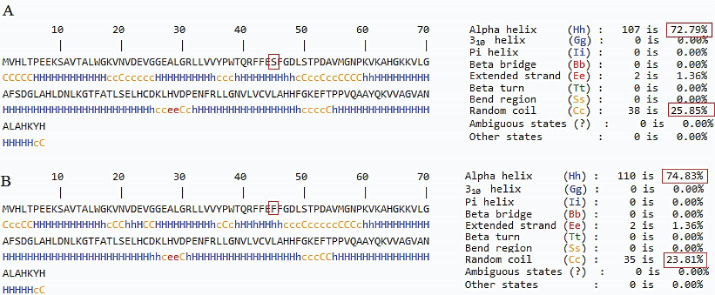
Changes in Secondary Structure Caused by Our Novel Variation. (A) Wild type protein secondary structure. (B) Mutant type protein secondary structure.

Table 2 provides a summary of the bioinformatics prediction scores for this variant. These scores represent the pathogenic status of this new variation in all except the mutation taster database. The patient’s parents made no contribution to the present study. The Iranome database had no such variation. Figure 2 presents a schematic representation of the beta globin protein and demonstrates the location of the change in the individual under the influence of the novel variant. Figure 3 also clearly indicates that the resulting change falls in the interspecies conserved domains. Finally, Figure 4 depicts the secondary structure of the beta-globin protein.

**Table 2:** Bioinformatics Prediction Scores for HBB: p.S44F Variant

**Software**	**Predict SNP**	**MAPP**	**PhD-SNP**	**PolyPhen-1**	**PolyPhen-2**	**SIFT**	**SNAP**	**MutationTaster**
Score	65% Del	41% Del	86% Del	59% Del	73%Del	53% Del	56% Del	Polymorphism

## Discussion

 There are over 280 mutations that affect the β-globin gene, resulting in a phenotype of β-thalassemia; a vast majority are point mutations in certain regions of the beta-globin gene that are important in terms of function.^2^ The clinical and laboratory findings are the basis for defining the three classifications of β-thalassemia: β-thalassemia minor, also referred to as carrier, is the heterozygous state that has no symptoms and manifests as mild anemia. Homozygosity or compound heterozygosity for β-thalassemia mutations result in more severe forms known as β-thalassemia intermedia and β-thalassemia major.^6^ The C > T novel reported variation (HbNL) at codon 44 of the β-globin gene changes a serine to a phenylalanine, which is a polar nucleophile amino acid with a non-polar aromatic amino acid substitution. Serine is a hydroxyl amino acid which is usually regarded as a hydrophile residue due to its hydroxyl group hydrogen bonding capacity. Based on its biochemical features, serine prefers to be situated on the protein surface.^7^ On the other hand, phenylalanine is a nonpolar amino acid and prefers to be deeply located within the protein hydrophobic core.^8^ As demonstrated in Figure 4, the serine to phenylalanine substitution causes less alpha helix and a more random coil structure. These changes in the secondary structure may result in tertiary structure changes and protein malfunction. This novel amino acid change occurring at the same position of previous pathogenic variation has been named hemoglobin Mississippi (HbMS: β44ser > cys). HbMS in the heterozygous form was clinically and hematologically normal and had mild microcytic anemia, but in compound heterozygous with β^+^-thalassemia showed all features of thalassemia intermedia.^9^ Accordingly, HbNL was clinically and hematologically normal with mild microcytic anemia, but according to the ACMG guideline, it cannot be assumed to be pathogenic.

 Based on *in silico* documents, the position of the reported variant, and hematological indices, it can be concluded that this variant falls into the nonpathogenic category, but should be considered when inherited in the compound heterozygous form with β-thalassemia (β^0^-thal) carrier, which might result in the phenotype of thalassemia intermedia. This finding has also helped expand the spectrum of β- thalassemia mutations found in Iran.

## References

[1] Farashi S, Harteveld CL (2018). Molecular basis of α-thalassemia. Blood Cells Mol Dis.

[2] Hamid M, Nejad LD, Shariati G, Galehdari H, Saberi A, Mohammadi-Anaei M (2017). The First Report of a 290-bp Deletion in β-Globin Gene in the South of Iran. Iran Biomed J.

[3] Khodaei GH, Farbod N, Zarif B, Nateghi S, Saeidi M (2013). Frequency of thalassemia in Iran and Khorasan Razavi. Int J Pediatr.

[4] Rezaee AR, Banoei MM, Khalili E, Houshmand M. Beta-Thalassemia in Iran: new insight into the role of genetic admixture and migration. Sci World J 2012;2012. 10.1100/2012/635183 PMC3539370 23319887

[5] MWer S, Dykes D, Polesky H (1988). A simple salting out procedure for extracting DNA from human nucleated cells. Nucleic acids Res.

[6] Hamid M, Zargan Nezhad E, Keikhaei B, Galehdari H, Saberi A, Sedaghat A (2021). Two novel and five rare mutations in the non coding regions of the β-globin gene in the Iranian population. Hemoglobin.

[7] Jindal R, Singla M, Kumar H (2015). Transport behavior of aliphatic amino acids glycine/l-alanine/l-valine and hydroxyl amino acids l-serine/l-threonine in aqueous trilithium citrate solutions at different temperatures. J Mol Liq.

[8] Loladze VV, Ermolenko DN, Makhatadze GI. Thermodynamic consequences of burial of polar and non-polar amino acid residues in the protein interior. J Mol Biol 2002;320(2):343-57. 10.1016/S0022-2836(02)00465-5 12079391

[9] Steinberg MH, Adams JG, Morrison WT, Pullen DJ, Abney R, Ibrahim A, et al. Hemoglobin Mississippi (β^44^ser- > cys) Studies of the Thalassemic Phenotype in a Mixed Heterozygote with β^+^-Thalassemia. J Clin Invest 1987;79(3):826-32. 10.1172/JCI112890 PMC424211 2434529

